# Influence of Controlled Cooling in Bimodal Scaffold Fabrication Using Polymers with Different Melting Temperatures

**DOI:** 10.3390/ma10060640

**Published:** 2017-06-11

**Authors:** Hernan Lara-Padilla, Christian Mendoza-Buenrostro, Diego Cardenas, Aida Rodriguez-Garcia, Ciro A. Rodriguez

**Affiliations:** 1 Escuela de Ingeniería y Ciencias, Tecnológico de Monterrey, Monterrey 64849, Mexico; A00815384@itesm.mx or hvlara@espe.edu.ec (H.L.-P); christian.mendoza@itesm.mx (C.M.-B); diego.cardenas@itesm.mx (D.C.); 2 Departamento de Ciencias de la Energía y Mecánica, Universidad de las Fuerzas Armadas ESPE, Sangolquí 171-5-231B, Ecuador; 3 Instituto de Biotecnología, Facultad de Ciencias Biologicas, Universidad Autónoma de Nuevo León, San Nicolas de los Garza 66455, Mexico; aidrod@hotmail.com

**Keywords:** tissue engineering, bone, bimodal scaffolds, fused deposition modeling, electrospinning, hybrid manufacturing process

## Abstract

The combination of different materials and capabilities to manufacture at several scales open new possibilities in scaffold design for bone regeneration. This work is focused on bimodal scaffolds that combine polylactic acid (PLA) melt extruded strands with polycaprolactone (PCL) electrospun fibers. This type of bimodal scaffold offers better mechanical properties, compared to the use of PCL for the extruded strands, and provides potential a means for controlled drug and/or growth factor delivery through the electrospun fibers. The technologies of fused deposition modeling (FDM) and electrospinning were combined to create 3D bimodal constructs. The system uses a controlled cooling system allowing the combination of polymers with different melting temperatures to generate integrated scaffold architecture. The thermoplastic polymers used in the FDM process enhance the mechanical properties of the bimodal scaffold and control the pore structure. Integrated layers of electrospun microfibers induce an increase of the surface area for cell culture purposes, as well as potential in situ controlled drug and/or growth factor delivery. The proposed bimodal scaffolds (PLA extruded strands and PCL electrospun fibers) show appropriate morphology and better mechanical properties when compared to the use of PCL extruded strands. On average, bimodal scaffolds with overall dimensions of 30 × 30 × 2.4 mm^3^ (strand diameter of 0.5 mm, strand stepover of 2.5 mm, pore size of 2 mm, and layer height of 0.3 mm) showed scaffold stiffness of 23.73 MPa and compression strength of 3.85 MPa. A cytotoxicity assay based human fibroblasts showed viability of the scaffold materials.

## 1. Introduction

Advanced strategies in bone tissue engineering and regenerative medicine have introduced the concept of in situ tissue regeneration, demanding scaffold designs with optimized physical properties and desirable performance. A potential approach to improve scaffold functionality is combining materials and manufacturing techniques [1]. The clinical success of scaffold-based approaches for bone regeneration relies on overcoming the limitations associated with single-phase biomaterials by developing synergistic combinations of biomaterials and emerging manufacturing technologies [2]. In the repair and regeneration of bone tissue, scaffolds require high elastic modulus (scaffold stiffness) in order to be retained in the bone defect space. An interconnected macropore structure is also required to enhance the diffusion and transportation of nutrients [3].

To overcome the current limitations, a recent approach has been to study multiscale scaffolds in an attempt to mimic hierarchical tissue structures [4]. Responding to these new requirements, advanced scaffold manufacturing processes are applied enabling architectural constructs designed with defined macro-, micro- and nanostructure [5]. Nano- and microporosity increases the surface area for cell attachment. This feature intensifies protein adsorption, which leads to an increase in cell attachment. In contrast, macroporous biomaterials provide less surface area, but offer a larger open volume, which enhances tissue ingrowth [6]. The scaffold chemistry, architecture, porosity, and rate of degradation should allow easy handling, provide an adequate mechanical environment, and facilitate cell attachment, cell proliferation, cell migration, waste–nutrient exchange, vascularization, and tissue growth [7].

The combination of additive manufacturing process and electrospinning has been targeted to make bimodal scaffolds for bone tissue applications, including structures at macro-, micro- and nanoscale [8,9]. Giannitelli et al. define a bimodal scaffold as the combination of some additive manufacturing process and electrospinning at the fabrication level to generate a fully integrated architecture, with application primarily in bone tissue regeneration [10]. Bimodal scaffolds usually contain macropores, with electrospun fibers providing suitable micro- and nanostructures for cell adhesion through increasing the surface area available for penetrating cells [11].

Table 1 summarizes the most relevant studies related to bimodal scaffolds that combine melt extrusion and electrospinning. In all cases of previous work, the extruded polymer has the same or a lower melting temperature compared to the material for electrospun fibers. There are a number of studies that use polycaprolactone (PCL) for both the strand and fiber processing.

As shown in the literature review, the range of scaffold designs is expanded as the manufacturing technology offers new materials and scale combinations. For the case of bimodal scaffolds using melt extrusion and electrospinning, there are particular benefits in using polymers with higher melting temperature for the extruded strands such as polylactic acid PLA (melting temperature of 180 °C) vs. polycaprolactone PCL (melting temperature of 90 °C). Bimodal scaffolds with PLA extruded strands could have better mechanical properties compared to PCL. The possibility of having an integrated architecture that includes PCL electrospun fibers opens design possibilities for several kinds of in situ controlled drug and/or growth factor delivery.

In the research reported here, bimodal scaffolds are generated focusing on the challenge of generated an integrated architecture based on PLA extruded strands and PCL electrospun fibers (see Table 1). The significant difference in melting temperatures between the extruded and the electrospun polymer produces unstable scaffold structures. Therefore, a controlled cooling system is introduced to improve the hybrid manufacturing process. These improved scaffolds were studied from the perspective of morphology and mechanical properties. In addition, an attempt is made to better understanding of the manufacturing process through simulation.

The previous work shown in Table 1 includes a wide range of scaffold designs and manufacturing parameters. Given these variations, comparison of mechanical properties and other features is difficult among different studies. PCL strands and fibers are commonly used to generate bimodal scaffolds (see Table 1). In order to validate the proposed approach, using PLA extruded strands and PCL electrospun fibers, scaffolds with PCL strands and fibers were used as reference in the study reported here.

## 2. Materials and Methods

### 2.1. Polymers

Commercial 3D printing filaments based on PLA and PCL were used to manufacture the polymer strands and to produce tensile specimens. The PLA used in this research work was ESUN 3D FILAMENT PLA with 1.75 mm of filament diameter with a nominal density of 1.240 g/cm^3^, melting temperature ~180 °C and a glass transition temperature ~60 °C. PLA is a biocompatible and resorbable aliphatic polyester [21]. The PCL used in this study was ESUN 3D FILAMENT eMORPH with 1.75 mm of filament diameter with a density of 1.145 g/cm^3^, melting temperature ~90 °C and a glass transition temperature of about ~60 °C. PCL is a biodegradable polyester [22]. For clarity, PCL used for fused deposition modeling strands will be referred to as PCLS throughout the paper. Polycaprolactone (PCL, Mn 80,000, Sigma-Aldrich, St Louis, MO, USA) was used to fabricate electrospun microfibers (melting temperature of ~60 °C). The polymer solution for electrospinning was prepared dissolving 10 wt % of PCL in acetone as solvent stirring at 35 °C for 8 h. The solution was injected through a blunt nozzle (20G) using 10 mL plastic syringes.

### 2.2. Scaffold Design and Processing

**Figure 1:**
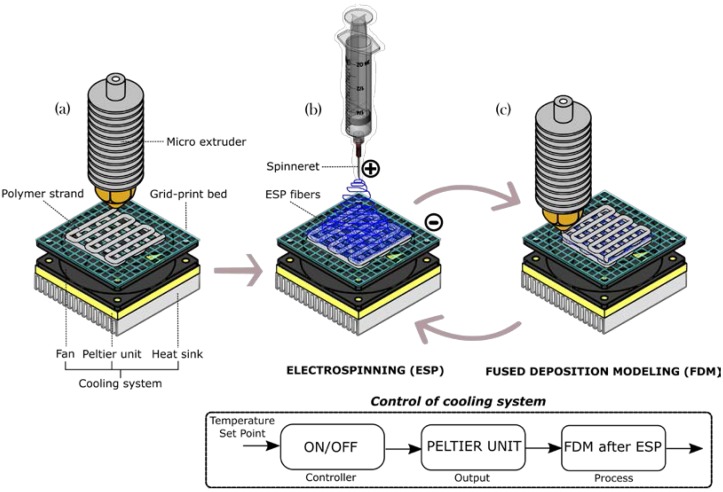
Schematic of hybrid processing with Fused Deposition Modeling (FDM) and electrospinning (ESP) with cooling system: (**a**) initial fused deposition modeling stage; (**b**) subsequent electrospinning stage; and (**c**) subsequent fused deposition modeling stage. The cooling control system was configured as open-loop system with an on/off controller. A constant temperature of 10 °C was setting as set point.

A special hybrid manufacturing process was used for the fabrication of scaffolds from the thermoplastic filaments, in combination with electrospun fibers (see Figure 1). A microextruder (similar process to Fused Deposition Modeling, FDM) with a diameter nozzle of 0.4 mm was used to plot the thermoplastic polymer. In addition, an integrated electrospinning system was used to generate layered micro fibers. First, a filament of thermoplastic polymer is placed through the feeder mechanism of the FDM system. Then, the polymer is melted in the microextruder to plot thermoplastic strands and complete the first two layers (see Figure 1a). After the first two layers, the scaffold is transferred to the electrospinning station (Figure 1b). In this fashion, a sequence of layers is deposited (two layers of FDM strands and one layer of electrospun fiber).

The bimodal scaffold overall design is prismatic. Nominal design values are the following: strand diameter (φ) of 0.5 mm, strand stepover (g) of 2.5 mm, pore size (g_p_) of 2 mm, and layer height (d) of 0.3 mm.

In order to test the effect of part cooling in this hybrid process, two groups of materials and processing conditions were used (see Table 2). For the Type A group, the FDM polymer is PLA, while PCLS was used for the Type B group. In all cases, the electrospun fibers are based on a PCL solution. The process parameters used for FDM and ESP are shown in Table 3.

The FDM nozzle temperature was set at 200 °C for PLA. Similarly, for PCLS, a working temperature of 120 °C for the FDM nozzle was found to generate an adequate deposition [14]. The room temperature was fixed at 20 °C.

The system is equipped with a controllable cooling system based on a Peltier thermopile, used to increase the cooling rate, which in turn avoids melting of electrospun microfibers. This cooling system (set to 10 °C) is the key to maintaining the integrity of both FDM strands and electrospun fibers, particularly when using FDM materials with higher melting temperature compared to the electrospun materials. A metallic grid-print bed was adapted to the cooling system, working simultaneously as platform for FDM deposition and as a collector to electrospinning process (Figure 1).

**Figure 2:**
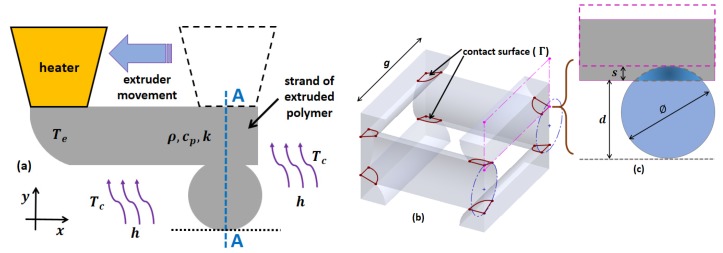
The model considered in the analysis of self-adhesion: (**a**) 2D representation of fused deposition; (**b**) unit cell considered as fundamental part of the scaffolds; and (**c**) 2D model considered for modeling.

The cooling system is activated simultaneously as a melted FDM layer is deposited on an electrospun mat. Once the platform moves to the ESP process, the cooling system is turned off. Thus, the cooling time depends on the time required to complete two layers of FDM strands above the electrospun mat. Figure 2 shows the model considered to study the influence of the cooling temperature on the self-adhesion process of strands.

The electrospun layers were made using 17 kV with a power supply (Model PS375, Stanford Research Systems, Inc., Sunnyvale, CA, USA) to produce the electric field. The flow rate of the electrospinning solution was fixed at 12 mL/h using a syringe pump (Model KD S200, KD Scientific, Boston, MA, USA). The distance between syringe and collector was fixed at 15 cm. The electrospinning deposition times on the top of the strands were fixed at 1.5 min. The G-code to manufacture the specimens was generated using a customized program developed in Matlab^®^.

### 2.3. Characterization of Scaffold Morphology

The ASTM F2450 Standard defines the dimension of a macropore/macroporosity structure > 100 μm, micropore/microporosity structure greater than 0.1 μm (100 nm) and less than about 100 μm (100,000 nm), and nanopore/nanoporosity includes sizes less than 100 nm (0.1 μm) [23]. The morphological characterization allows observing the scaffold cross-section and surface through Scanning Electron Microscope EVOMA25 (Carl Zeiss, Oberkochen, Germany). SEM images allowed examining the internal geometry of the scaffold and measuring the geometrical parameters considered in its design: pore size (g_p_), strand diameter (φ), layer height (d), and the diameter distribution of the electrospun PCL microfibers inside the bimodal scaffold. The final shape of the bimodal scaffolds was a square prism. All dimensions were measured using the software Image J (imagej.nih.gov, Bethesda, MD, USA).

The apparent macroporosity (Π) of the scaffolds was obtained by following equation:

(1) $$\Pi =1-\frac{\rho_{m}}{\rho_{s}}$$

where $\rho_{m}$ and $\rho_{s}$ represent the apparent density of the scaffold and the density of the solid (bulk density), respectively [24]. The apparent density of the scaffold was calculated dividing the weight by overall volume for the scaffold. The bulk density of PLA and PCLS was measured considering five cubes of 1 cm per side with 100% infill. Weight measurements were conducted with an analytical balance (Mettler Toledo AE 240). The average bulk density for PLA and PCLS was 1.12 g/cm^3^ and 1.02 g/cm^3^, respectively. To calculate the macroporosity of basic and bimodal scaffolds, five specimens of each group were weighed. Pore size was assumed to be the distance between strands and was determined by the software ImageJ using images from optical microscopy (DISCOVERY V8 Carl Zeiss) and SEM.

The fiber diameter distribution of the PCL electrospun microfibers was calculated using ImageJ software. Measurements were made at 100 random positions. Diameter distribution and average diameter was computed and reported, following the method proposed by Augustine et al. [25].

### 2.4. Characterization of Scaffold Mechanical Properties

The mechanical properties of the scaffolds were evaluated by measuring the scaffold stiffness (E) and scaffold yield strength (*σ_y_*). The mechanical properties assessment was performed in dry state of the scaffolds under ambient conditions at 20 °C. All compression tests were carried out using prismatic scaffolds (30 × 30 × 2.4 mm^3^) at the rate of 0.5 mm/min with an Instron^®^ universal test bench, with a load cell of 5 kN (Instron Model 3300, Nordwood, MA, USA). The apparent stress was estimated based on the total projected area the prismatic scaffold. The strain was determined as the ratio between the scaffold height variation and the scaffold initial height.

### 2.5. Process Simulation

The self-adhesion phenomenon is a major factor in thermoplastic polymer processing such as thermal extrusion, welding, and injection molding [14]. The resulting contact surface represents has important influence on the mechanical properties and porosity of the scaffold. In FDM, a cooling process produces the self-adhesion phenomenon, immediately after the biopolymer is deposited. The analysis of the cooling process and self-adhesion of extruded polymer strands was conducted considering section A-A (Figure 2a). In this case, the finite element method (FEM) was used to predict the cooling of a strand from a temperature above the melting point, under the influence of the cooling temperature ($T_{c}$). To simplify the problem, the following assumptions were considered: (a) the polymer melt is deposited fast enough above the base layer; and (b) the viscous heating effect is neglected. Hence, as an initial condition, a constant temperature throughout the considered domain $T_{e}$ is assumed.

A 2D approximation to the self-adhesion model was considered with the temperature as a function T(x,y,t) of two spatial variables (x,y) and the time variable t. The heat equation is the following:

(2) $$\rho c_{p}\frac{\partial T}{\partial t}-\nabla \left(k\nabla T\right)=\dot{q}$$

where ρ is the density, $c_{p}$ is the specific heat, k is the thermal conductivity, and $\dot{q}$ is the heat flux [26]. The scheme used for solving (2) was the ω-family approximation:

(3) $$\left(\left[M^{e}\right]+\Delta t\omega \left[K^{e}\right]\right){\left\{T\right\}}^{i+1}=\left(\left[M^{e}\right]-\Delta t\left(1-\omega \right)\left[K^{e}\right]\right){\left\{T\right\}}^{i}+\Delta t\left(\omega {\left\{f\right\}}^{i+1}+\left(1-\omega \right){\left\{f\right\}}^{i}\right)$$

where $\left[K^{e}\right]$ is the stiffness matrix, $\left[M^{e}\right]$ is the mass matrix, {f} is the load vector, Δt is the interval time, and ω=0.5 (Crank–Nicholson scheme).

### 2.6. Cytotoxicity Assay

In the functionality of bimodal scaffolds, the FDM strands act as a mechanical support for bone ingrowth, while the electrospun fibers provide an adequate environment for cellular activities and potential drug delivery functions. Then, the cytotoxicity test of Type B bimodal scaffolds with cooling was used to demonstrate the viability of the electrospun mats.

To assess the in vitro cytotoxicity of the bimodal scaffolds, the International ISO 10993-5 was followed [27]. Samples of Type B bimodal scaffolds with cooling of 5 × 5 mm^2^ (n = 9) were soaked in 70% ethanol for 30 min, followed by washing twice in sterile PBS. Then, the samples were exposed to UV light for 15 min, 30 min prior to cell seeding. Human fibroblasts (Detroit 548 ATCC, CCL-116, American Type Culture Collection, Rockville, MD, USA) were placed into sterile of 96-well plates with in low glucose Dulbecco’s Modified Eagle’s Medium (DMEM, GIBCO, Invitrogen, Grand Island, NY, USA) supplemented with 10% FBS (Laboratorios Microlab, Mexico City, Mexico) and 1% penicillin–streptomycin (Sigma, St. Louis, MO, USA). DMEM were harvested and seeded on each wall with the scaffolds samples at a density of 4000 cells per well in a total volume of 200 μL of complete media. The cultures were kept at 37 °C and 5% CO_2_ in an incubator (Sanyo, MCO-19AIC, (UV), Moriguchi, Japan). Replacement of the culture medium was changed every day and the cells were trypsinized upon reaching a confluence of 50%.

Cell proliferation was assessed using the MTT assay proposed by [28]. This method is based on the conversion of MTT into formazan, which determines mitochondrial activity. Fibroblasts were seeded, in triplicates, in a 96-well culture microtiter plates at a density of 3 × 10^3^, in triplicate. One hundred microliters of medium without cells and 100 μL of 1% Triton X-100 in PBS were added to the wells and were used as blank and positive control, respectively. The microtiter plates were incubated during 48 h in an incubator with 5% CO_2_ at 37 °C. After incubation, 20 μL of MTT were added to the test wells and allowed to react for 4 h at 37 °C and 5% of CO_2_. The supernatant was carefully removed from each well. One hundred microliters of isopropanol was added to each well and resuspended until all crystals of blue formazan were dissolved. Absorbance of the dye was measured by reading the absorbance at 570 nm on a Biotek Synergy 2 microplate reader (Winooski, VT, USA).

## 3. Results

### 3.1. Scaffold Morphology

**Figure 3:**
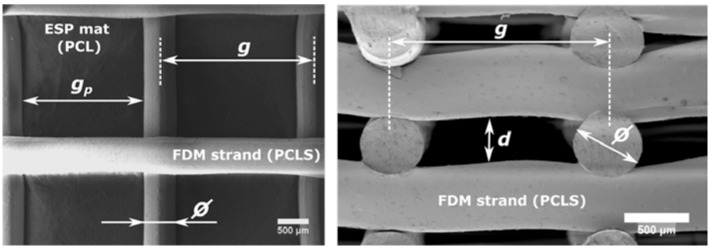
SEM micrographs: (**a**) Top section view of Type B scaffold (FDM + ESP + Cooling); and (**b**) cross section view of Type B scaffold (FDM + ESP + Cooling).

Figure 3 shows a sample of the bimodal scaffold. Table 4 summarizes the scaffold morphology and mechanical properties (to be discussed in detail in the next section). Actual strand diameter has a systematic error producing larger dimensions compared to the nominal value (nominal strand diameter (φ) of 0.5 mm). Actual pore size is consistently smaller than the target value (nominal pore size (g_p_) of 2 mm). Despite these systematic errors, in terms of scaffold morphology, the cooling system does not have a statistically significant influence.

**Figure 4:**
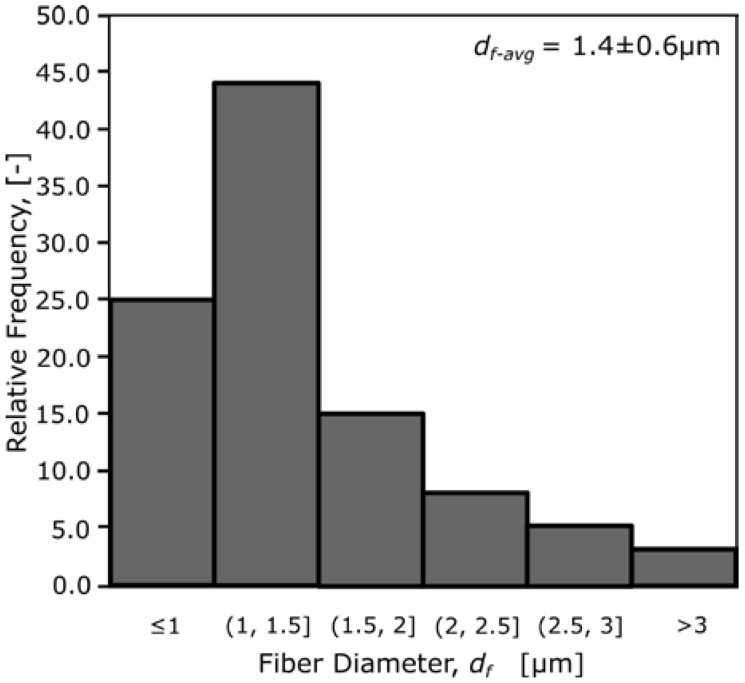
Electrospun polycaprolactone (PCL) mat characterization.

**Figure 5:**
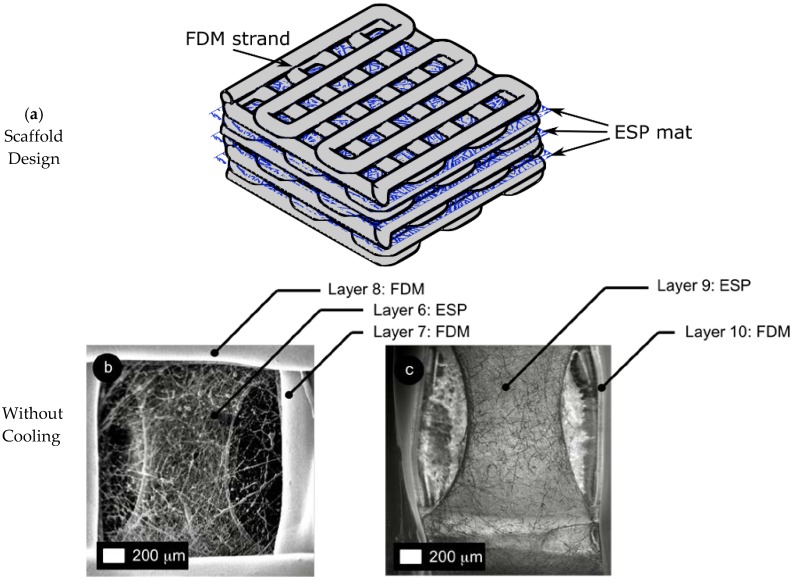

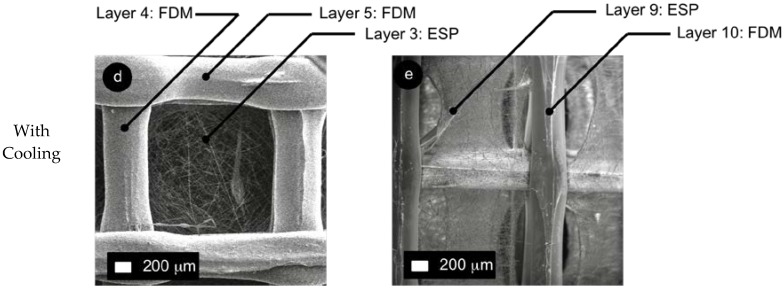
Influence of cooling system on scaffold morphology with 11 layers for scaffold Type A with polylactic acid (PLA) strands electrospun polycaprolactone (PCL) microfibers: (**a**) scaffold design with 11 layers; (**b**,**c**) electrospun mat morphology without cooling; and (**d**,**e**) electrospun mat morphology with cooling.

Figure 4 shows the distribution of fiber diameter for the electrospun PCL mat. Average fiber diameter is 1.4 μm. Figure 5 shows the effect of cooling on the scaffold morphology for Type A scaffolds. The samples shown in Figure 5 are designed with total of 11 layers (i.e., three sets of two FDM layers + one ESP layer and two additional FDM on top, see Figure 5a).

For the condition without cooling, there is partial melting of electrospun fibers and a peeling effect (i.e., separation of the electrospun mat from the FDM strands) (see Figure 5b). Layer 9 (third electrospun mat) shows the formation of a film once the molten fibers get closer together (see Figure 5c). These adverse effects on the electrospun mats are due to the considerable difference between melting temperature of the electrospun PCL (60 °C) and the temperature generated in the microextruder nozzle (200 °C for PLA).

When the cooling system is on, the integrity of the electrospun mesh is improved and the peeling effect is reduced. Layer 3 (first electrospun mat) shows complete integrity of the fibers and no peeling effect (see Figure 5d). Once the scaffold progresses, layer 9 (third electrospun mat) shows some peeling effect (see Figure 5e).

For the Type B scaffolds, the peeling effect is not present for the condition without cooling because the same basic polymer is being used for strands (PCLS) and fibers (PCL). However, some partial melting is observed.

### 3.2. Scaffold Mechanical Properties

**Figure 6:**
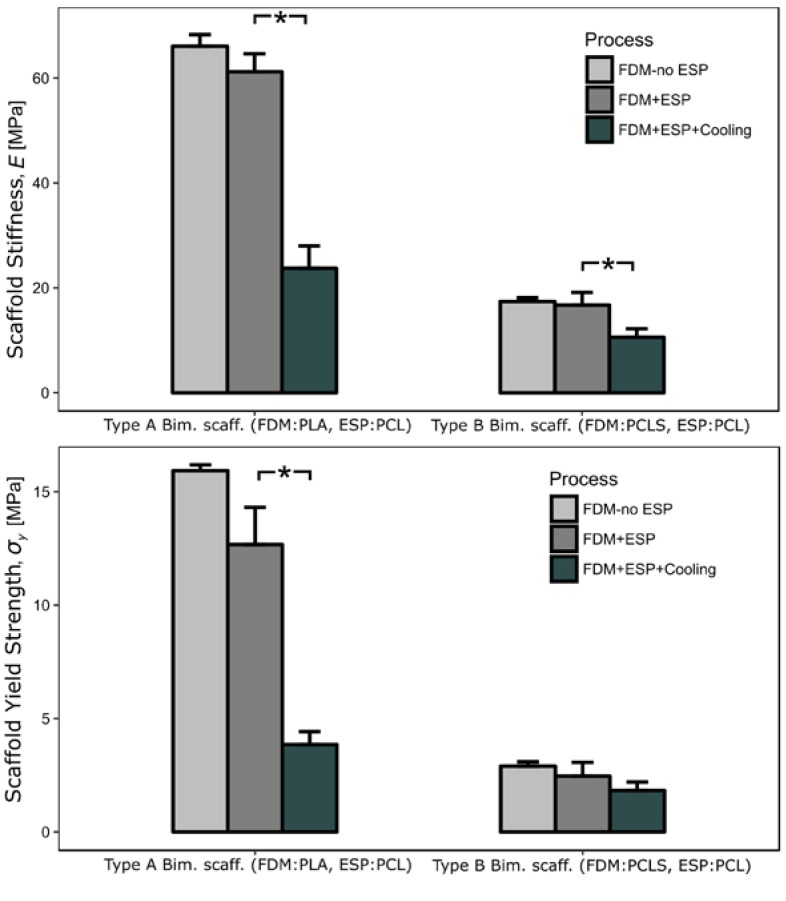
Mechanical properties of bimodal scaffolds (scaffold stiffness and scaffold yield strength). Note: * means *p*-value < 0.05.

Figure 6 shows the mechanical properties of the bimodal scaffolds. The elastic zone for the tensile tests was considered to evaluate the values for the scaffold stiffness and scaffold yield strength. The overall failure mechanism is delamination between the FDM strands.

For the Type A bimodal scaffolds, the use of electrospun fibers does not have a statistically significant effect on their mechanical properties. However, the cooling system that improves the integrity of the electrospun mesh does have a significant influence on the mechanical properties. The scaffold stiffness drops from an average of 61.23 to 23.73 MPa (standard deviations are indicated in Table 4). A significant drop in strength from 12.67 to 3.85 MPa is also observed for the Type A bimodal scaffolds with cooling (standard deviations are indicated in Table 4).

Similarly to Type A bimodal scaffolds, Type B bimodal scaffolds do not show statistically significant changes in mechanical properties when FDM and ESP are combined. For Type B bimodal scaffolds, the use of the cooling system only affects the scaffold stiffness, with statistical significance. Some more in-depth analysis into the failure mechanism of delamination was performed with process simulation, to be reported in the following section. While the scaffold stiffness and the scaffold yield strength decrease dramatically when cooling Type A scaffolds, these properties are higher than those of Type B scaffolds.

### 3.3. Process Simulation

The numerical simulations for analysis of the self-adhesion phenomenon were implemented with a multiphysics software (Elmer, www.csc.fi, Espoo, Finland) using FEM. The self-adhesion phenomenon was represented as two perpendicular strands in contact. The 2D computational model was created using triangle elements, and wire discretization algorithm with a fineness of 0.01 mm. For the numerical computation of the self-adhesion surface, a transient analysis was considered to establish the necessary time for cooling the junction area. The material thermal properties considered for simulation purposes were PLA and PCLS (Table 5).

The temperature field and its change with time is useful to establish the effective region of adhesion, which, in turn, has an influence on the mechanical failure by delamination of the scaffold layers. Based on extensive experimentation, to adequately extrude the thermoplastic strands, the functional range of the processing temperature was determined to be 200 °C for PLA and 120 °C for PCLS. These temperature intervals ensure an adequate viscosity of the molten polymers and appropriate adhesion between layers.

**Figure 7:**
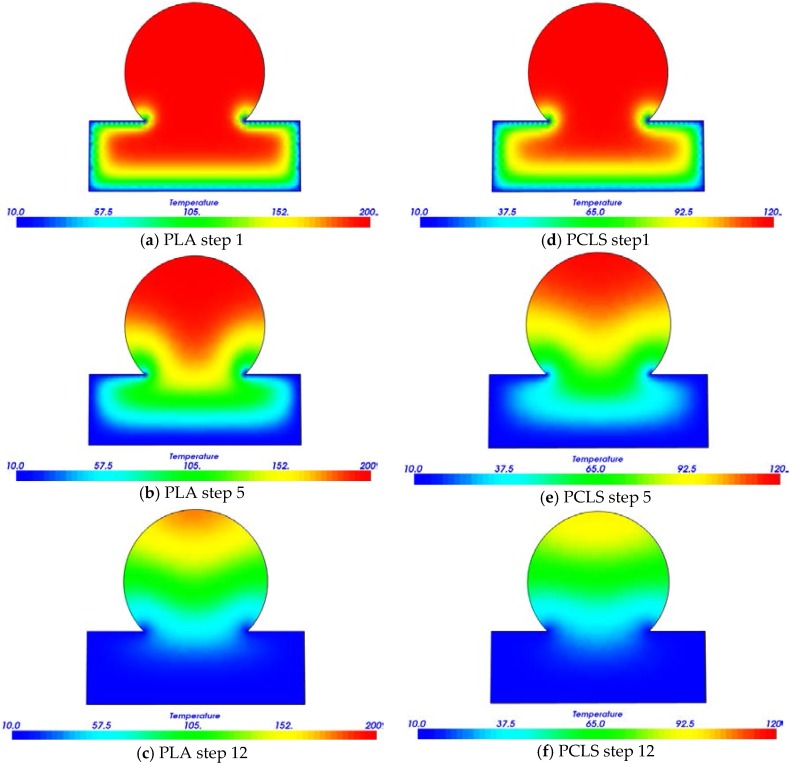
Thermal distribution during the self-adhesion process at different steps for: PLA (**a**–**c**); and PCLS; (**d**–**f**) (temperature units are in °C). The step size is equal to 0.05 s.

Figure 7 shows the process simulation at section A-A in Figure 2a. The temperature field as a function of time is shown for PCLS and PLA. At the adhesion surface, PLA simulation shows a temperature of approximately 150 °C (see Figure 7b), while PCL is around 70 °C. Step 5 corresponds to a cooling time of 0.25 s. Therefore, after 0.25 s from initial conditions, the adhesion surface is already below the melting temperature in both cases. Therefore, the adhesion time (period when both previous and new strand have contact with temperatures above the melting point) should be approximately between 0.10 and 0.15 s.

### 3.4. Cytotoxicity Test

**Figure 8:**
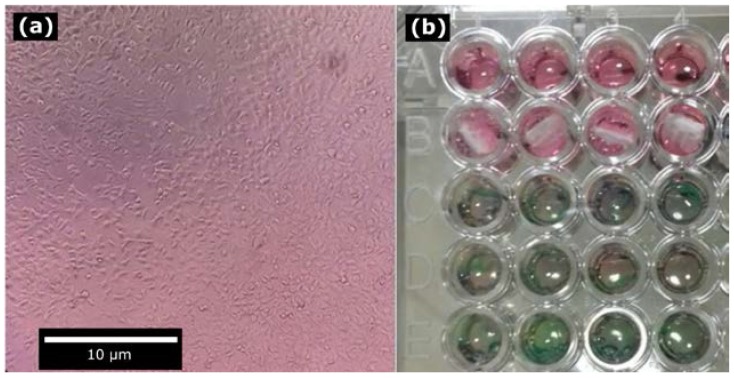
Cytotoxicity test in agreement with ISO 10993-5: (**a**) fibroblast confluency; and (**b**) cytotoxicity test of Type B bimodal scaffolds with cooling.

Our data revealed that all scaffolds show viability higher than 88% (see Figure 8), which confirms that Type B bimodal scaffolds with cooling presented cytocompatibility (cell viability higher than 50%).

## 4. Discussion

The combination of fused deposition modeling and electrospinning with polymers of different melting point was shown to be a viable hybrid process with the aid of controlled cooling. The use of the cooling system allows the combination of PLA strands with a higher melting temperature compared to the PCL electrospun mesh, with the known benefits in potential delivery of various growth factors and/or drugs for bone regeneration. The main significant drawback of the proposed approach is the reduction in the mechanical properties of bimodal scaffolds when controlled cooling is used.

### 4.1. Scaffold Processing

FDM strands are limited in terms of encapsulation of drugs, growth factors, or other thermosensitive agents due to the inherent high temperature of the process. In contrast, the electrospinning technique has been widely used for drug delivery and to enhance the cell attachment. 

The developed cooling system reported here was the first approximation in order to preserve the integrity of electrospun fibers when they are embedded into a scaffold matrix made with PLA or PCLS strands using the FDM process. The cooling system generates a gradient temperature along the height (thickness) of the bimodal scaffold, with a constant temperature of 10 °C at the lower layer. At this stage in the development, the distribution of temperature during the manufacturing of the scaffolds was not measured. Further characterization of the temperatures profile is required through a non-invasive technique, such as thermographic imaging.

### 4.2. Scaffold Morphology

In general, the optimal pore size for bone tissue regeneration has been a topic of debate [15]. Thavornyutikarn et al. (2014) suggest that a pore size between 300 and 500 μm allows cells to penetrate into the pore structure and promote new bone formation, as well as vascularization [29]. This pore size range enhances the diffusion rates of a scaffold, transportation of the nutrients and by-products but is not sufficient for large scaffold volumes [3]. The pore size used in the present work to demonstrate the proof-of-concept was aimed at 2 mm, which is too large in comparison with the recommendations. However, this parameter and others can be easily set to reach custom-made scaffolds.

The partial melting of fibers and film generated in Type A scaffolds without cooling reduces the capacity of scaffolds to delivery drugs and/or growth factors. These morphological changes in the fibers indicate an aggressive high temperature that could degrade the chemical compounds embedded as part of the electrospinning process.

For Type A scaffolds (PLA strands + PCL fibers) with cooling, some degree of peeling phenomenon appears in the upper layers of the microfiber mats scaffolds. However, in general, electrospun mats showed a uniform distribution of the microfibers, and a suitable microporosity, that increases available surface area for initial cell attachment and proliferation [11].

The configuration of the cooling system does not allow keeping a constant cooling temperature in the whole scaffold structure. Thus, a steady temperature of 10 °C can be guaranteed at the first FDM layer of the scaffold. Given the relatively low heat conductivity of polymers, it is expected that a temperature gradient will be created as the subsequent layers are manufactured. This temperature gradient might be responsible for the appearance of some peeling for Type A scaffolds in Layer 9 (ESP) with cooling (see Figure 5e).

Currently, imaging of the peeling effect was conducted in selected regions of the scaffold for a qualitative analysis of tendencies. In order to completely characterize the electrospun mat integrity in terms of *x*-*y* plane distribution, a more comprehensive study is required with detailed imaging of all pores in the structure and measurements of the proportion of peeling relative to the complete projected area of a given pore. Additional characterization of temperature distribution in the *x*-*y* plan is also required.

### 4.3. Scaffold Mechanical Properties

Kim et al. report a compressive modulus (scaffold stiffness) of 15.0 MPa for a bimodal scaffold (PCL strands and fibers) with 60% interconnected porosity [13]. Lee et al. report a Young’s modulus (scaffold stiffness) of 9.5 MPa with 56% of interconnected porosity (PCL strands and collagen fibers) [15]. Compared against these previous studies, the bimodal scaffolds reported here show better mechanical properties (scaffold stiffness of 23.7 MPa with 60% interconnected porosity) when using PLA strands and PCL fibers (see Table 4).

The significant reduction in the scaffold stiffness of PLA with the same geometrical configuration could be a consequence of the change in the polymer crystallinity due to the thermal processing conditions [30]. Carrasco et al. reported an increase of the elastic modulus and an increase in yield strength after an annealing treatment of PLA samples initially processed by injection molding and extrusion. The rapid cooling of the polymer during this kind of processing limits the crystallization process and produces changes in the mechanical properties [31].

The phenomenon reported by Carrasco et al. could be the underlying mechanism for the reduction in scaffold stiffness of Type A scaffolds processed under cooling conditions. The cooling system produces a faster temperature reduction after microextrusion, with a potential reduction in crystallinity of the final polymer.

Skoglund and Fransson showed that the crystallization temperature can drop in polycaprolactone from 47 to 27 °C, as the cooling rate increases from 0.31 to 40 °C/min [32]. Therefore, for the Type B scaffolds (based on PCLS strands), the reduction in mechanical properties for processing under cooling could be also derived from crystallinity kinetics.

For the scaffolds Type A with PLA extruded strands under cooling conditions, the bonding was poor and then the premature failure by delamination was evident under compression tests. The high temperature gradient produced by the cooling system of these bimodal scaffolds strands is caused by a poor adhesion between layers, and therefore an easier mechanical failure by delamination. The high-temperature gradient shortens the adhesion time and therefore produces a weaker union between strands. Furthermore, the reduction of the scaffold yield strength can be explained by the combination of both delamination and crystallinity, as previously reported [31].

### 4.4. Process Simulation

More detailed study of the heat transfer conditions is required in order to optimize the current hybrid manufacturing process. An important boundary condition in further simulation studies is to consider a realistic pore size between 300 and 500 μm.

### 4.5. Cytotoxicity Test 

A wide range of biodegradable polymer materials and scaffolding fabrication techniques for bone tissue engineering have been investigated [33]. Several in vitro and in vivo tests have been carried out successfully, demonstrating that polymers like PLA and PCL can fulfill the standard ISO 10993-5.

The results indicate that the microfibers mats in the bimodal scaffold can provide a suitable surface to maintain adhesion of the injected cells. The cytotoxicity assay does not reveal whether fibroblasts penetrated the electrospun microfiber mats within the scaffold. However, the microfiber mats are layered on top of FDM strands, so it is assumed that the cells can easily infiltrate from the side area of the scaffolds. Further studies are required to validate the penetration of the seeded cells into the bimodal scaffolds.

### 4.6. Potential Applications

There is a growing necessity to develop new scaffold manufacturing techniques to improve the functionality in bone regeneration applications. Recent interest in bimodal and multiphasic scaffolds for studies in periodontal and large bone defects requires improved control over how such structures are fabricated. The use of synthetic polymers for bone tissue engineering has grown considerably through advances in polymer synthesis technologies, especially with regards to controlled radical polymerization and scaffolding methods [2].

From a clinical perspective, the tissue-engineered bone graft should allow for a mechanically secure and stable fixation of the host tissue [3]. Currently, the process reported here for bimodal scaffolds has been studied as a proof-of-concept and initial in vitro testing. However, the manufacturing approach presented here has potential applications in maxillofacial procedures, such as alveolar bone and sinus augmentation, where in situ drug delivery is desired in order to counter the natural tendency for infections in oral treatments.

## 5. Conclusions and Future Work

The proposed approach to generate bimodal scaffolds based on PLA extruded strands and PCL electrospun microfibers has been shown to be feasible. This approach represents a technological improvement of an already existing additive manufacturing process. The introduction of the cooling system allows consistency of the integrated architecture that combines extruded strands and meshes of electrospun fibers. The current approach is able to overcome the difference in melting temperature between the polymers used for extrusion and electrospinning. The use of controlled cooling also produces a reduction of the mechanical properties for the PLA-based bimodal scaffolds. However, even these diminished mechanical properties are still higher than to those of PCLS-based scaffolds.

The proposed future work is to continue optimization of the system in order to achieve better mechanical properties in bimodal scaffolds with PLA extruded strands and PCL electrospun microfibers. Additional characterization of the temperature field and its influence on the integrity of the electrospun mats is also proposed.

## Figures and Tables

**Table 1:** Related works for bimodal scaffolds combining melt extrusion and electrospinning.

Extruded Strand Processing		Electrospun (ESP) Fiber Material	Reference
Process	Material		
Melt extrusion with screw dispensing	PCL	PLGA	[12]
Melt extrusion with pressure assisted dispensing	PCL	PCL	[13]
	PCL	PCL and PCL + COLL	[14]
	PCL	COLL	[15]
	PCL + starch	PCL	[16]
	PCL + HA	PU	[17]
	PEOT + PBT	PEOT + PBT	[18]
	PEOT + PBT	PEOT + PBT	[19]
	GEL + ALG	PCL	[20]
Fused Deposition Modeling (FDM)	PLA	PCL	Current study
	PCLS	PCL	

ALG = Sodium alginate, COLL = Collagen, GEL = Gelatin, PBT = poly-(butylene terephthalate), PCL = Polycaprolactone, PCLS = Polycaprolactone (Commercial FDM filament), PEOT = poly (ethylene oxide terephthalate), PLA = Polylactic acid, PLGA = poly (lactic-co-glycolic acid), PU = Polyurethane.

**Table 2:** Materials and cooling conditions.

Groups	FDM + No ESP	FDM + ESP	FDM + ESP with Cooling
Type A	PLA	PLA + PCL	PLA + PCL
Type B	PCLS	PCLS + PCL	PCLS + PCL

**Table 3:** Process parameters for the manufacturing of bimodal scaffolds.

**Fused Deposition Modeling (FDM)**	**PLA**	**PCLS**
Filament feed speed (f) [mm/min]	60	60
Axis linear speed (v) [mm/min]	120	120
Deposition temperature $\left(T_{e}\right)$ [°C]	200	120
Lay-down pattern orientation [°]	0/90	0/90
Cooling system temperature $\left(T_{c}\right)$ [°C]	10	10
**Electrospinning (ESP)**	**PCL**	
Voltage $\left(V_{elp}\right)$ [kV]	17	
Flow rate $\left(Q_{elp}\right)$ [mL/h]	12	
Overall processing time $\left(t_{p}\right)$ [min] ***	18	

*** Note: size of 30 × 30 × 2.4 mm^3^ with three electrospun layers.

**Table 4:** Morphology and mechanical properties of bimodal scaffolds.

Bimodal Scaffold		Strand Diameter, φ [μm] (n = 10) +	Pore Size, *g_p_* [μm] (n = 10) +	Porosity, Π [%] (n = 5) ++	Scaffold Stiffness, *E* [MPa] (n = 3) ++	Scaffold Yield Strength, *σ_y_* [MPa] (n = 3) ++
Type A PLA-PCL	FDM-no ESP	540 ± 22	1940 ± 51	60.1 ± 5.6	66.06 ± 2.24	15.93 ± 0.25
	FDM + ESP	536 ± 10	1933 ± 21	61.2 ± 1.5	61.23 ± 3.41	12.67 ± 1.64
	FDM + ESP + Cooling	515 ± 30	1896 ± 36	59.9 ± 4.2	23.73 ± 4.27	3.85 ± 0.57
Type B PCLS-PCL	FDM-no ESP	540 ± 28	1955 ± 44	60.7 ± 3.2	17.42 ± 0.70	2.90 ± 0.19
	FDM + ESP	541 ± 16	1948 ± 37	61.8 ± 1.8	16.74 ± 2.38	2.46 ± 0.61
	FDM + ESP + Cooling	531 ± 24	1953 ± 48	62.2 ± 2.9	10.58 ± 1.62	1.83 ± 0.37

Note: + replications are based on measurements in different regions of the scaffold. ++ replications are based on several scaffolds.

**Table 5:** Thermal properties of the biopolymers used in the self-adhesion modeling.

Property	PLA	PCLS
Density (ρ) [g/cm^3^]	1.250	1.100
Thermal conductivity (k) [W/m·K]	0.13	0.21
Specific heat $\left(c_{p}\right)$ [J/kg·K]	200	120

## Nomenclature

**Symbol**	**Description**
$c_{p}$	Specific heat [J/kg·K]
d	Layer height [μm]
$d_{f}$	Fiber diameter [μm]
E	Scaffold stiffness [MPa]
f	Filament feed speed [mm/min]
{f}	Load vector
g	Strand stepover [μm]
$g_{p}$	Pore size [μm]
k	Thermal conductivity [W/m·K]
$\left[K^{e}\right]$	Stiffness matrix
$\left[M^{e}\right]$	Mass matrix
$\dot{q}$	Heat flux [W/m^2^]
$Q_{elp}$	Flow rate [mL/h]
s	Overlap [μm]
T	Temperature [°C]
$T_{c}$	Cooling system temperature [°C]
$T_{e}$	Deposition temperature [°C]
t	Time [s]
$t_{p}$	Overall processing time [min]
v	Axis linear speed [mm/min]
$V_{elp}$	Voltage [kV]
Π	Apparent macroporosity [%]
ρ	Density [g/cm^3^]
$\rho_{m}$	Apparent density [g/cm^3^]
$\rho_{s}$	Bulk density of the polymer strand [g/cm^3^]
$\sigma_{y}$	Yield strength [MPa]
ϕ	Diameter of a polymeric strand [μm]
ω	Crank–Nicholson factor

## References

[1] Park S., Lee B., Kim M., Lee S., Yoo J., Atala A. (2016). Situ Tissue Regeneration: Host Cell Recruitment and Biomaterial Design.

[2] Shrivats A., McDermott M., Hollinger J. (2014). Bone tissue engineering: State of the union. Drug Discov. Today.

[3] Hutmacher D.W. (2000). Scaffolds in tissue engineering bone and cartilage. Biomaterials.

[4] Srinivasa S., Jayakumar R., Chennazhi K., Levorson E., Mikos A., Nair S., Jayakumar R., Nair S. (2012). Biomedical Applications of Polymeric Nanofibers.

[5] Gong T., Xie J., Liao J., Zhang T., Lin S., Lin Y. (2015). Nanomaterials and bone regeneration. Bone Res..

[6] Holzapfel B., Reichert J., Schantz J., Gbureck U., Rackwitz L., Nöth U., Jakob F., Rudert M., Groll J., Hutmacher D. (2013). How smart do biomaterials need to be? A translational science and clinical point of view. Adv. Drug Deliv. Rev..

[7] Ma P. (2008). Biomimetic materials for tissue engineering. Adv. Drug Deliv. Rev..

[8] Ivanovski S., Vaquette C., Gronthos S., Hutmacher D., Bartold P. (2014). Multiphasic scaffolds for periodontal tissue engineering. J. Dent. Res..

[9] Woodruff M., Hutmacher D. (2010). The return of a forgotten polymer—Polycaprolactone in the 21st century. Prog. Polym. Sci..

[10] Giannitelli S., Mozetic P., Trombetta M., Rainer A. (2015). Combined additive manufacturing approaches in tissue engineering. Acta Biomater..

[11] Dalton P., Vaquette C., Farrugia B., Dargaville T., Brown T., Hutmacher D. (2013). Electrospinning and additive manufacturing: converging technologies. Biomater. Sci..

[12] Mota C., Puppi D., Dinucci D., Errico C., Bártolo P., Chiellini F. (2011). Dual-Scale Polymeric Constructs as Scaffolds for Tissue Engineering. Materials.

[13] Kim G., Son J., Park S., Kim W. (2008). Hybrid process for fabricating 3D hierarchical scaffolds combining rapid prototyping and electrospinning. Macromol. Rapid Commun..

[14] Park S., Kim T., Kim H., Yang D., Park T. (2008). Development of dual scale scaffolds via direct polymer melt deposition and electrospinning for applications in tissue regeneration. Acta Biomater..

[15] Lee H., Yeo M., Ahn S., Kang D., Jang C., Lee H., Park G., Kim G. (2011). Designed Hybrid Scaffolds Consisting of Polycaprolactone Microstrands and Electrospun Collagen-Nanofibers for Bone Tissue Regeneration. J. Biomed. Mater. Res. Part B Appl. Biomater..

[16] Martins A., Chung S., Pedro A., Sousa R., Marques A., Reis R., Neves N. (2009). Hierarchical starch-based fibrous scaffold for bone tissue engineering applications. J. Tissue Eng. Regen. Med..

[17] Kang Y., Shin J., Park S., Kim Y., Gu S., Wu Y., Ban H., Shin J. (2015). A three-dimensional hierarchical scaffold fabricated by a combined rapid prototyping technique and electrospinning process to expand hematopoietic stem/progenitor cells. Biotechnol. Lett..

[18] Moroni L., Schotel R., Hamann D., de Wijn J., van Blitterswijk C. (2008). 3D Fiber-Deposited Electrospun Integrated Scaffolds Enhance Cartilage Tissue Formation. Adv. Funct. Mater..

[19] Nandakumar A., Barradas A., de Boer J., Moroni L., van Blitterswijk C., Habibovic P. (2013). Combining technologies to create bioactive hybrid scaffolds for bone tissue engineering. Biomatter.

[20] Yu Y., Zheng L., Chen H., Chen W., Hu Q. (2014). Fabrication of hierarchical polycaprolactone/gel scaffolds via combined 3D bioprinting and electrospinning for tissue engineering. Adv. Manuf..

[21] Patrício T., Domingos M., Gloria A., Bártolo P. (2013). Characterisation of PCL and PCL/PLA Scaffolds for Tissue Engineering. Procedia CIRP.

[22] Ebersole G., Buettmann E., MacEwan M., Tang M., Frisella M., Matthews B., Deeken C. (2012). Development of Novel Electrospun Absorbable Polycaprolactone (PCL) Scaffolds for Hernia Repair Applications. Surg. Endosc..

[23] ASTM International (2010). Standard Guide for Assessing Microstructure of Polymeric Scaffolds for Use in Tissue Engineered Medical Products.

[24] Zein I., Hutmacher D., Tan K., Teoh S. (2002). Fused deposition modeling of novel scaffold architectures for tissue engineering applications. Biomaterials.

[25] Augustine R., Malik H., Singhal D., Mukherjee A., Malakar D., Kalarikkal N., Thomas S. (2014). Electrospun polycaprolactone/ZnO nanocomposite membranes as biomaterials with antibacterial and cell adhesion properties. J. Polym. Res..

[26] Huang H. (2013). Finite Element Analysis for Heat Transfer.

[27] ISO (1993). Biological Evaluation of Medical Devices. Part 5: Tests for Cytotoxicity: In Vitro Methods.

[28] Mosmann T. (1983). Rapid colorimetric assay for cellular growth and survival: application to proliferation and cytotoxicity assays. J. Immunol. Methods.

[29] Thavornyutikarn B., Chantarapanich N., Sitthiseripratip K., Thouas G., Chen Q. (2014). Bone tissue engineering scaffolding: computer-aided scaffolding techniques. Prog. Biomater..

[30] Garlotta D. (2001). A Literature Review of Poly(Lactic Acid). J. Polym. Environ..

[31] Carrasco F., Pagès P., Gámez-Pérez J., Santana O., Maspoch M. (2010). Processing of poly(lactic acid): Characterization of chemical structure, thermal stability and mechanical properties. Polym. Degrad. Stab..

[32] Skoglund P., Fransson A. (1996). Continuous cooling and isothermal crystallization of polycaprolactone. J. Appl. Polym. Sci..

[33] Holland T., Mikos A. (2005). Review: Biodegradable polymeric scaffolds. Improvements in bone tissue engineering through controlled drug delivery. Tissue Eng. I.

